# Dead on arrival in a low-income country: results from a multicenter study in Pakistan

**DOI:** 10.1186/1471-227X-15-S2-S8

**Published:** 2015-12-11

**Authors:** Munawar Khursheed, Junaid Ahmad Bhatti, Fatima Parukh, Asher Feroze, Syed Saad Naeem, Haseeb Khawaja, Junaid Abdul Razzak

**Affiliations:** 1 Department of Emergency Medicine, Aga Khan University, Karachi, Pakistan; 2 Sunnybrook Health Sciences Centre Research Institute, Toronto, Ontario, Canada; 3 Institute for Clinical Evaluative Sciences, Toronto, Ontario, Canada; 4 University of Toronto, Department of Surgery, Toronto, Ontario, Canada; 5 Johns Hopkins International Injury Research Unit, Department of International Health, Johns Hopkins Bloomberg School of Public Health, Baltimore, MD, USA; 6 Department of Emergency Medicine, Shifa International Hospital, Islamabad, Pakistan; 7 Department of Emergency Medicine, John Hopkins School of Medicine, Baltimore, MD, USA; 8 The author was affiliated with the Department of Emergency Medicine, Aga Khan University, Karachi, Pakistan at the time when study was conducted

**Keywords:** Critical illness, cardiopulmonary resuscitation, prehospital care, surveillance

## Abstract

**Background:**

This study assessed the characteristics of dead on arrival (DOA) patients in Pakistan.

**Methods:**

Data about the DOA patients were extracted from Pakistan National Emergency Department Surveillance study (Pak-NEDS). This study recruited all ED patients presenting to seven tertiary care hospitals during a four-month period between November 2010 and March 2011. This study included patients who were declared dead-on-arrival by the ED physician.

**Results:**

A total of 1,557 DOA patients (7 per 1,000 visits) were included in the Pak-NEDS. Men accounted for two-thirds (64%) of DOA patients. Those aged 20-49 years accounted for about 46% of DOA patients. Nine percent (n = 72) of patients were brought by ambulance, and most patients presented at a public hospital (80%). About 11% of DOA patients had an injury. Factors significantly associated (p < 0.05) with ambulance use were men (adjusted odds ratio [aOR] = 2.72), brought to a private hospital (OR = 2.74), and being injured (aOR = 1.89). Cardiopulmonary resuscitation (CPR) was performed on 6% (n = 42) of patients who received treatment. Those brought to a private hospital were more likely to receive CPR (aOR = 2.81).

**Conclusion:**

This study noted a higher burden of DOA patients in Pakistan compared to other resourceful settings (about 1 to 2 per 1,000 visits). A large proportion of patients belonging to productive age groups, and the low prevalence of ambulance and CPR use, indicate a need for improving the prehospital care and basic life support training in Pakistan.

## Background

Premature, preventable deaths from severe injuries and critical illnesses are a major health burden globally [1,2]. Between 10% and 50% of these deaths occur before reaching hospitals [3,4]. The burden of these deaths is disproportionately high in low- and middle-income countries (LMICs) [4-6], and is explained by the pervasive health risks and under-resourced healthcare systems [2]. More often than not, the causes of preventable deaths are neglected in LMICs, especially if they occurred before reaching or just after arriving at a health facility [7-9]. Studying the underlying cause of these deaths in high-income countries (HICs) has greatly facilitated the implementation of interventions to prevent premature deaths [1,9-12].

The literature lacks a consensus-based definition of deaths before reaching a facility [4,9]. This imprecision results from patients without vital signs who are pronounced dead after receiving some care in the emergency department (ED) [9]. A common practice in some EDs is to define the above cases as DOA or dead on arrival, which introduces the risk of misclassification [8,9,13,14]. Nevertheless, the broad definition of DOA may include patients who were either declared dead upon arrival to an emergency department with no resuscitation attempt or those who died after failed resuscitation, usually within the first 15 to 60 minutes of arrival [8,9,15].

Care at the prehospital and emergency department levels play a pivotal role in reducing deaths, especially DOA, from injuries and critical illnesses [2,3,16,17]. The prevalence of DOA is usually less than 1 to 2 per 1,000 ED visits in settings with well-established, resourceful emergency care systems [9,18]. In resource-poor settings, the prevalence of DOA is expected to be higher, but few studies are available about the burden and characteristics of DOA patients in these settings [17,19]. Single-center studies from Pakistan, a low-income country with a population of over 180 million, suggested that DOA contributed significantly to ED deaths [19-21]. The generalizability of these findings was limited however because almost all of these studies were conducted in resourceful, private healthcare settings. This study assessed the characteristics of DOA patients presenting in diverse healthcare settings in Pakistan.

## Methods

### Setting and design

The data for this study were extracted from a larger surveillance study conducted in Pakistan, the Pakistan National Emergency Departments Surveillance Study or Pak-NEDS [22]. The main aims of Pak-NEDS were piloting an ED-based surveillance in Pakistan and profiling the ED burden of disease and care at the level of major tertiary care facilities in Pakistan. The study duration was from November 2010 to March 2011 and it recruited patients presenting at seven high-volume EDs of teaching hospitals: Aga Khan University (AKU) and Jinnah Post-graduate Medical Center (JPMC) in Karachi; Benazir Bhutto Hospital (BBH) in Rawalpindi; Lady Reading Hospital (LRH) in Peshawar; Mayo Hospital (MHL) in Lahore; Sandeman Provincial Hospital in Quetta; and Shifa International Hospital (SIH) in Islamabad (See [22] for details). AKU and SIH were private hospitals, and the rest were public hospitals. AKU was the coordinating center for the study. Ethical approval of the study conduct was obtained from ethical review committees of all participating hospitals.

### Data collection and measures

A standard data collection tool was used to collect information about all patients. This tool, which included information about up to three presenting complaints and up to seven treatments provided at the ED, was developed from two sources: 1) National Hospital Ambulatory Medical Care Survey tool of the Centers for Disease Control and Prevention, United States (US) [23]; and 2) previous emergency care evaluations in Pakistan [24,25]. The tool was finalized by expert opinions and was pilot tested at the Department of Emergency Medicine, AKU.

Trained research assistants who were hired for this study collected the data. They worked in three shifts ensuring 24-hour data collection. Emergency care-related information such as presenting complaints and treatments were documented from admission sheets (issued at the ED), ED registers, and medico-legal registers (where applicable). Patient-specific information that may have been missed in the official records such as residence or mode of transport (e.g., ambulance) was collected from patients or from those who brought them to the ED. Hard copies of the data collection tool were sent to the coordinating center at AKU. Trained data entry operators entered the data on spreadsheets at AKU by using EpiInfo version 3.3.2. A research supervisor coordinated the data entry and randomly verified about 5% of the entries for accuracy.

### Patient selection

Pak-NEDS included DOA as a presenting complaint. All patients who were labelled as DOA by ED physicians were included. For these cases, information extracted from the larger study included demographic characteristics such as age and gender as well as the ED care, e.g., hospital, mode of arrival, presenting complaints, injury (if reported), care providers (e.g., nurses, physicians), imaging, and treatment (if administered).

### Analyses

The characteristics of DOA patients were described using proportions. Patients' ages were re-categorized as ten-year strata. Rates per 1,000 visits for all patients and for different strata were computed. These rates have been used in resourceful settings to describe the ED care of patients with specific diagnoses [26,27]. Associations of ambulance use (prehospital care) or cardiopulmonary resuscitation between the different groups were assessed using a chi-square test and multivariate logistic regression models. Final models included significantly associated variables (*p *≤ 0.05). All analyses were performed using SPSS version 19.

## Results

There were 1,557 (0.6%) patients recorded as DOA in the Pak-NEDS. Males accounted for the majority of patients (64.0%, n = 970). About half (46.3%, n = 699) of the patients belonged to productive age groups (20-49 years). Patients aged 50-59 years accounted for 16.9% of patients. Four out of five patients were brought to a public hospital (80.2%, n = 1,249). Less than ten percent of all patients (n = 72, 8.9%) were brought in an ambulance (Table 1).

**Table 1:** Characteristics of dead on arrival patients presenting to seven emergency departments in Pakistan (November 2010 to March 2011)

	N	%	Per 1,000 visits
Total	1557	100	6.5
Gender			
- Male	970	64	6.6
- Female	548	36	6.0
Age (in years)			
0 to 9	58	3.8	6.7
10 to 19	98	6.5	2.9
20 to 29	304	20.1	4.1
30 to 39	166	11.0	2.9
40 to 49	229	15.2	5.3
50 to 59	256	16.9	11.2
50 to 69	206	13.6	18.4
70+	194	12.8	25.9
Type of hospital			
Public hospital	1249	80.2	5.6
Private hospital	308	19.8	20.5
Ambulance use			
- Yes	72	8.9	8.2
- No	739	91.1	3.3

The average rate of DOA among presenting complaints was 6.5 per 1,000 visits. The DOA rate per 1,000 visits was higher in those aged 50 to 59 years (11.2), 60-69 years (18.4), 70 years or more (25.9), in those brought to a private hospital (20.5), and in those brought in an ambulance (8.2).

The common presenting complaints in DOA patients were fever (10.9%), followed by chest pain (6.3%, n = 98), abdominal pain (5.6%, n = 87), generalized pain (4.6%, n = 71), and diarrhea (3.3%, n = 51) (Table 2). Of the reported patients, 10.7% (n = 165) came to the ED because of an injury. Three quarters of the injury patients (73.5%, n = 108) reported unintentional injuries. Road traffic injuries accounted for just over a third of injuries (36.4%, n = 28), followed by burns (22.1%, n = 17), and falls (14.3%, n = 11).

**Table 2:** Emergency care characteristics of dead on arrival patients presenting to seven emergency departments in Pakistan (November 2010 to March 2011)

	N	%
Presenting complaints		
- Fever	168	10.9
- Chest pain	98	6.3
- Abdominal pain	87	5.6
- Generalized pain	71	4.6
- Diarrhea/loose motions	51	3.3
- Any injury	165	10.7
Injury		
- Unintentional	108	73.5
- Intentional	39	26.5
Mechanism of injury		
- Road traffic injury	28	36.4
- Burns	17	22.1
- Falls	11	14.3
- Others (n≤5)	21	27.2
- Other	38	49.3
Seen by		
- Nurse	511	56.2
- Paramedic	393	43.2
- House physician	377	41.4
- Medical officer	625	68.7
- Resident	213	23.4
- Consultant physician	103	11.3
Imaging		
- X-ray	304	95.6
- Computerized tomography scan	5	1.0
- Ultrasound	12	3.8
Treatment		
- Cardiopulmonary resuscitation	42	6.0
- Central venous line/intravenous fluids	140	19.9

Upon arrival, most patients were seen by nurses (56.2%, n = 511) or paramedics (43.2%, n = 393). Medical officers (physicians with complete practice privileges) saw over two-thirds of patients (68.7%, n = 625) and consultants were involved in 11.3% (n = 103) of patients. About half of the patients received some treatment (45.1%, n = 703). Cardiopulmonary resuscitation (CPR) was performed on 6.0% (n = 42) of these patients. Other treatments administered to patients recorded as DOA included analgesia (75.6%), antibiotics (38.5%), and intravenous fluids (19.9%).

Age, gender, type of hospital brought to, and being injured were all significantly associated (*P*<0.05) with ambulance use in the univariate analysis (Table 3). The multivariate logistic regression analysis showed that compared to those aged 70 years or more, DOA cases aged 0-9 years, 10-19 years, 30-39 years, 40-49 years, and 50-59 years were less likely to use ambulance services. When compared to women, men were almost three times more likely to use ambulance services (adjusted odds ratio [aOR]= 2.72). The likelihoods of ambulance use were higher in those brought to a private hospital (aOR = 2.74) or those who were injured (aOR = 1.89) as compared to other patients.

**Table 3:** Ambulance use in dead on arrival patients in seven emergency departments in Pakistan (November 2010 to March 2011)

	Ambulance used		Ambulance not used		P	Odds ratio	95% Confidence Interval
					
	N	%	n	%			
Age (in years)					0.04		
0 to 9	4	5.7	39	5.4		0.47	0.13-1.76
10 to 19	4	5.7	72	9.9		0.20	0.05-0.72
20 to 29	28	40.0	210	28.9		0.45	0.18-1.13
30 to 39	7	10.0	106	14.6		0.28	0.09-0.84
40 to 49	5	7.1	111	15.3		0.18	0.05-0.61
50 to 59	8	11.4	108	14.9		0.29	0.09-0.88
50 to 69	5	7.1	45	6.2		0.63	0.18-2.16
70+	9	12.9	36	5.0		1	
Gender					0.001		
- Male	57	11.3	447	88.7		2.72	1.40-5.28
- Female	13	4.3	289	95.7		1	
Type of hospital					0.08		
- Public	47	65.3	552	74.7		1	
- Private	25	34.7	187	25.3		2.74	1.50-5.01
Injury					0.004		
- No	44	66.7	575	81.4		1	
- Yes	22	33.3	131	18.6		1.89	1.05-3.38

Age, type of hospital brought to and being injured were all significantly associated with CPR in the univariate analysis (Table 4). The multivariate regression analysis showed patients brought to a private hospital were more likely to have CPR performed on them than those brought to a public hospital (aOR = 2.82).

**Table 4:** Cardiopulmonary resuscitation (CPR) in dead on arrival patients in seven emergency departments in Pakistan (November 2010 to March 2011)

	CPR done		CPR not done		P	Odds ratio	95% Confidence Interval
					
	N	%	n	%			
Age (in years)					0.01	*	
0 to 9	8	16.7	50	3.4		2.71	0.98-7.52
10 to 19	2	4.2	96	6.6		0.46	0.10-2.18
20 to 29	11	22.9	293	20.0		0.70	0.28-1.73
30 to 39	4	8.3	162	11.1		0.49	0.15-1.25
40 to 49	4	8.3	225	15.4		0.38	0.11-1.25
50 to 59	5	10.4	251	17.2		0.47	0.15-1.43
50 to 69	5	10.4	201	13.7		0.58	0.19-1.76
70+	9	18.8	185	12.6		1	
Gender							
- Male	35	71.4	935	63.6			
- Female	14	28.6	534	36.4			
Type of hospital					<0.001		
- Public	30	57.7	1219	81.0		1	
- Private	22	42.3	286	19.0		2.81	1.53-5.12
Injury					0.01		
- No	26	60.5	643	78.1			
- Yes	17	39.5	180	21.9			
Ambulance					0.54		
- No	25	86.2	714	91.3			
- Yes	4	13.8	28	8.7			

* Age was significantly associated (P = 0.02) with CPR use in the multivariate logistic regression analysis

## Discussion

This multicenter ED surveillance study described the burden and characteristics of DOA patients in Pakistan. The study showed that the rates of DOA were 2 to 4 times higher than average in those aged 50 years or older and in those who were brought to a private hospital. Injuries accounted for one in nine DOAs; of these patients, three-quarters had unintentional injuries including road traffic injuries, burns, and falls [24,25,28]. Ambulance use was significantly higher for male patients, in those brought to a private hospital, and in injured patients than in other DOA patients. Similarly, the practice of CPR at ED was significantly higher for those brought to a private hospital as compared to those who were brought to a public hospital.

Some DOA patients may receive intense initial emergency care [9]. International statistics on DOA burden in EDs in resource-limited settings are mostly unavailable, and this study provides useful insights about the burden of DOA patients in Pakistani EDs [8,9,13-15,18,29,30]. This study showed that the proportion of DOA patients observed in this study was three times higher than in other regional countries, such as around 2 per 1,000 visits in Taiwan [18]. This study showed that DOA rates were higher than average in certain demographic categories for which it was not expected such as being middle-aged (50-59 years) [25,31]. While investigation of factors associated with DOA in this relatively younger age group is beyond the scope of this study, findings highlight the need for improving emergency care in specific population groups that are expected to have better outcomes [31].

This study may have prehospital and emergency care-related implications. First is the utilization of ambulance services, which was low in Pakistan [32]. Accessibility and affordability could be the two main barriers for the utilization of ambulance services. With the development of free-of-cost (or for nominal fees) ambulance services in Karachi and Lahore, it was expected that ambulance use in these cities would be higher, but an overall usage rate of less than ten percent suggested low accessibility of ambulance services. A surrogate for affordability of these services was the higher usage rate in those brought to a private hospital, as it is likely that these patients could afford and had access to these services. In the case of one private hospital - Shifa International Hospital - nearly three-quarters of patients arrived in ambulances (not shown separately above). In developed countries with established prehospital care systems, deployment of ambulances and paramedics are prioritized according to severity of the health problem [34]. Our findings indicated that ambulance use in severe cases such as DOAs in Pakistan was lower than the international standards [4,15]. These discrepancies highlight the need to improve accessibility to prehospital care in Pakistan. Of note, many philanthropic organizations have their own ambulances, but they do not have a single command and control center to coordinate response and widen their services areas [25,28]. A common dispatch center with predefined protocols of service delivery could be an important step towards improving the prehospital services in Pakistan [35].

This study also indicated that specific demographic and patient factors were associated with ambulance use in Pakistan [36]. For example, female DOA patients were less likely to use ambulance service as compared to male DOA patients. The role of men as breadwinners and because they were likely to be outside the home for work may be possible factors which could explain this gender disparity [37]. The study also showed that being injured increased the likelihood of ambulance service use in DOA cases compared to in those with other conditions [32]. It is likely that because these cases occurred on roads, accessibility increased the chances for ambulance use. The role of bystanders and the general public in alerting and calling for an ambulance has been largely ignored in low-income settings, including Pakistan [38,39]. Few initiatives exist in Pakistan to train bystanders in basic life support skills, and these findings advocate for the development and expansion of such initiatives to reduce disparities in ambulance use [40,41].

Another major finding of this study is the low rate of CPR use on those who presented as DOA to EDs [42]. In settings like the United States, the prevalence of CPR performance is very high in critically ill patients arriving at EDs (e.g., 108 of 140 cases in [14]). Our study showed that the patients brought to a private hospital were more likely to have CPR performed than other patients. The resuscitation training of medical graduates was not obligatory in Pakistan until very recently [41,43,44]. In Pakistan, and as noted in this study, physician care providers are mostly house physicians (new graduates with limited privileges for practice) and medical officers or residents (who have at least one year of experience and have complete privileges). It is likely that most of them have not received CPR training, and this may have contributed to the low rate of CPR use [41,26]. Similarly, not all staff members are trained in resuscitation skills, which could explain this low rate of CPR. Taken together, these findings suggest that the training and practice of CPR should be strongly encouraged and prioritized in Pakistan to provide optimal prehospital care to critically ill patients [43].

This study has several limitations. Firstly, a lack of predefined criteria and reliance on an ED physician's assessment of DOA may lead to misclassification [9]. Secondly, this study is unable to document the cause of death, which was the case in previous studies from Pakistan as well [19-21]. Thirdly, given the design of the study, it was impossible to collect in-depth information about the type and quality of prehospital care or CPR. Nonetheless, this study did provide useful direction for future research about the need to investigate DOA in specific populations, e.g., in children and middle-aged individuals.

## Conclusion

This multicenter study showed that DOA patients contributed significantly to the ED burden. Most DOA patients belonged to productive age groups. Overall low ambulance use and relatively higher use by men and private hospital patients suggested a need for improving access to ambulance services in Pakistan. Similarly, low CPR use in DOA patients suggested that more efforts are needed to train the general population and ED staff in basic life support.

## Competing interests

The authors declare that they have no competing interests.

## Authors' contributions

MK and JB wrote the initial manuscript. JB, MK, and AF performed the analyses. FP and SSN assisted in analysis and manuscript writing. HAK was local collaborator at Shifa International Hospital, Islamabad and provided critical review of the draft. JAR conceptualized Pak-NEDS, supervised data collections and entry on spreadsheets and provided critical review of this draft. All authors critically reviewed and approved the final manuscript before submission.
